# Dual Antimelanogenic Effect of Nicotinamide-Stabilized Phloretin Nanocrystals in Larval Zebrafish

**DOI:** 10.3390/pharmaceutics14091825

**Published:** 2022-08-30

**Authors:** Yixuan Li, Hong Xiang, Xinyue Xue, Yilan Chen, Zhiyuan He, Zhongrui Yu, Li Zhang, Xiaoqing Miao

**Affiliations:** 1 Marine College, Shandong University, Weihai 264209, China; 2 School of Chemistry & Chemical Engineering, Liaocheng University, Liaocheng 252059, China

**Keywords:** phloretin, nicotinamide, nanocrystals, anti-melanin, tyrosinase, zebrafish

## Abstract

Melanin is a kind of dark insoluble pigment that can cause pigmentation and free-radical clearance, inducing melasma, freckles, and chloasma, affecting the quality of life of patients. Due to poor water solubility and low safety, the absorption of poorly water-soluble drugs is limited by the hinderance of a skin barrier. Therefore, it is necessary to develop new, safe, and highly efficient drugs to improve their transdermal absorption efficiency and thus to inhibit the production of melanin. To address these issues, we developed a new nicotinamide (NIC)-stabilized phloretin nanocrystals (PHL-NCs). First, NC technology significantly increased the solubility of PHL. The in vitro release results indicated that at 6 h, the dissolution of the PHL-NIC-NCs was 101.39% ± 2.40% and of the PHL-NCs was 84.92% ± 4.30%, while that of the physical mixture of the two drugs was only 64.43% ± 0.02%. Second, NIC acted not only as a stabilizer to enlarge the storage time of PHL-NIC-NCs (improved to 10-day in vitro stability) but also as a melanin transfer inhibitor to inhibit melanin production. Finally, we verified the melanin inhibition effect of PHL-NIC-NCs evaluated by the zebrafish model. It showed that 0.38 mM/L PHL-NIC-NCs have a lower tyrosinase activity at 62.97% ± 0.52% and have less melanin at 36.57% ± 0.44%. The inhibition effect of PHL-NCs and PHL-NIC-NCs was stronger compared to the positive control arbutin. In conclusion, the combination of NIC and PHL achieves better inhibition of tyrosinase and inhibition of melanin production through synergism. This will provide a direction to the subsequent development of melanin-inhibiting drugs and the combined use of pharmaceutical agents.

## 1. Introduction

Melanin is a type of dark insoluble pigment that causes pigment precipitation and free-radical scavenging [1,2], inducing skin disorders, such as age spots, freckles, melasma, and malignant melanoma [3]. Existing drugs for treating hyperpigmentation can be divided into the following five categories [4]: cell receptor antagonists, inhibitors of melanocyte stimulation (retinoids), tyrosinase enzyme inhibitors (resveratrol) [5], inhibitors of melanosome transfer (niacinamide (NIC)) [6], and drugs that degrade melanin in keratinocytes (glycolic acid). However, there are a few problems with these drugs. First is the safety of the drugs itself. For example, kojic acid, hydroquinone, and corticosteroids lead to adverse reactions, such as dermatitis and skin irritation, ochronosis, cytotoxicity, and skin cancer [3]. Arbutin is degraded to toxic hydroquinone under high temperature, ultraviolet radiations, and a dilute acid [7]. L-ascorbic acid is heat-reactive and easy to degrade [8]. Second is low bioavailability [9]. Many melanogenesis inhibitors, including most flavonoids or polyphenols, such as ellagic acid [8], have excellent whitening and antioxidant effects. However, the brick-mud structure of the stratum corneum (SC) limits the transdermal absorption of these insoluble drugs. Thus, new, safe, and highly effective drugs are necessary, as well as formulations designed to enhance the accumulation of drugs in the epidermal basal layer and thus inhibit melanogenesis. Therefore, it is essential to design new drugs and their preparations in a safe and highly efficient manner to enhance their transdermal absorption efficiency, thus inhibiting the production of melanin.

Phloretin (PHL) is a new and highly effective drug that dilutes melanin and whitens the skin by inhibiting the tyrosinase activity (the core of melanin synthesis) during melanin synthesis [10]. In addition, some studies have found that PHL has low toxicity (PHL belongs to flavonoids), which has a good application prospect [11]. However, PHL is difficult for practical application because its poor water solubility and stability [12]. The solubility and skin permeability of PHL could be improved by nanocarrier technology, such as the preparation of nanoemulsions, lipid nanoparticles (NPs) [13], chitosan NPs [14], and polymer NPs [15]. However, the extensive use of carrier materials results in low drug loading, high production costs, and safety problems. Compared to nanocarriers, nanocrystals (NCs) have the advantages of higher drug loading (almost 100%), good stability, less toxic side effects, and easy preparation properties. In addition, NCs provide a range of benefits to cross the SC barrier, which include higher concentration gradients, follicular targeting, diffusion corona, and skin adhesion [16]. Therefore, NCs are an attractive strategy for improving the solubility of PHL and enhancing bioavailability. NIC is a recognized whitening agent that acts after melanin synthesis to inhibit the transfer of melanin from melanomas to surrounding keratinocytes and reduce melanin production in the skin [6,17].

The zebrafish model for melanin research is a hot topic in recent years. Numerous pigment cell and skin-equivalent models have also been described for evaluating the efficacy of melanogenic regulatory compounds. However, the data obtained from in vitro studies may not be directly extrapolated to the in vivo situation (physiological shortcomings). In addition, there are some economic correlation shortcomings, such as long time consumption and high cost. Therefore, in vivo tests using animal models or humans are the most physiologically relevant. While zebrafish has high genetic similarity with humans and has melanocytes and melanosomes similar to those in humans, it is often used as an important model for melanocyte-related studies, which has the advantages of small size, large sample size, and short test period [18]. Its body is transparent and has melanin on its surface, which makes it easy to observe and identify pigment cells during development, and one can simply observe the pigmentation process [19,20]. In addition, zebrafish can be used to study the percutaneous effects of drugs by taking advantage of the characteristics that zebrafish is percutaneously absorbed before 7 days post-fertilization (dpf). Zebrafish is extensively experimentally validated as a whole-animal model, which can study the inhibition of melanogenesis or the screening of stimuli. Experiments have demonstrated that zebrafish systems are novel alternatives to mammalian models [19,21,22]. For example, Choi et al. [19] tested the feasibility of zebrafish as a model system to determine the role of melanogenic stimulants. They used a-MSH (a major physiological stimulus of pigmentation) to induce the synthesis of eumelanin and investigated the effects of several compounds on zebrafish pigmentation. The results showed that zebrafish can also serve as a good model for the evaluation of melanogenic stimuli. In addition, during the developmental process of melanogenic regulatory compounds, potential toxicity should be considered. Zebrafish can be used to simultaneously determine drug toxicity on a biological basis. It is easier to determine whether drugs are toxic to organisms through mortality, morphological deformity assessment, and heart rate [19]. This cannot be achieved simultaneously by cell culture, pigment screening, enzyme analysis, and other experiments. In recent years, nanoformulations have also been used for zebrafish evaluation [23] and are ideal for nanoparticle and drug analysis using non-invasive techniques [24]. Therefore, it was ideal for the evaluation of the drugs in our experiment.

As the formation of melanin involves mainly two pathways, we assumed that combined inhibition of tyrosinase activity and melanin transfer may strongly increase the anti-hyperpigmentation effect. To prove this hypothesis, PHL-NCs stabilized by NIC were fabricated. Zebrafish embryos were used to evaluate the anti-melanin efficacy of PHL-NCs and PHL-NIC-NCs in this study.

## 2. Materials and Methods

### 2.1. Materials

PHL with purity higher than 98%, NIC (purity > 98%), HPMC, sodium deoxycholate, L-DOPA, and ethyl acetate were bought from Macklin (Shanghai, China). The chemical reagents in this article were chromatographic and of analytical grade.

### 2.2. Fabrication PHL-NIC-NCs

PHL-NCs were prepared by the anti-solvent precipitation method. Briefly, 0.5 mL of 20 mg/mL of PHL ethanol solution as the organic phase and 20 mg HPMC (PHL and HPMC mass ratio = 1:2) were added to 19.5 mL of water as the aqueous phase. Under 1000 rpm stirring, the organic phase was injected into the aqueous phase and the preparation was completed after 10 s stirring (85-2A Jingxin, Shanghai, China). The organic solvent was removed by overnight open placement.

Similarly, PHL-NIC-NCs were prepared. PHL and NIC (mole ratio = 1:1) were dissolved in ethanol as the organic phase, and HPMC was added to 19.5 mL of water as the aqueous phase. Under 1000 rpm stirring, the organic solution was injected into the aqueous solution and the preparation was completed after 10 s stirring (85-2A Jingxin, Shanghai, China). The organic solvent was removed by overnight open placement.

An aggregation-induced emission (AIE) probe 1,1,2,3,4,5-hexaphenylsilole (HPS) was used to hybridize the NCs. HPS-NCs and Hybridized HPS of PHL-NCs (PHL-HPS-NCs) and PHL-NIC-NCs (PHL-NIC-HPS-NCs), which were dissolved in ethyl acetate, were also prepared by the anti-solvent precipitation method, as described earlier. The HPS and PHL mole ratio was 1:10.

### 2.3. Investigation on the Stability of NCs

The particle size and polydispersity index (PDI) of PHL-NCs and PHL-NIC-NCs were measured by the Nano^®^ Zetasizer (Malvern Instruments, Worcestershire, UK) on days 1, 2, 3, 5, 7, 10, and 15. The suspensions were stored in a 4 °C refrigerator.

### 2.4. Characterization of NCs

The particle size and PDI of NCs were measured by the Nano^®^ Zetasizer (Malvern Instruments, Worcestershire, UK). The morphologies of the NCs were observed by a Nova Nano SEM (FEI, Hillsboro, OR, USA). The thermal properties of the raw medicine, stabilizer, physical mixture of the raw medicine and stabilizer, and three particle sizes of NCs were measured by a Diamond TG/DTA (Perkin Elmer, Waltham, MA, USA). The Raman spectra were obtained by a confocal Raman microscopy (inVia plus, Renishaw, UK). All these measurements were performed at three different times.

### 2.5. In Vitro Drug Release 

The release of PHL-NCs and PHL-NIC-NCs in vitro was studied by the dialysis method [25]. A mixture of PBS (pH 6.8) and ethanol (7:3, *w*/*w*) [13] was selected as the release medium. Next, 10 mg of drugs (physical mixture of raw medicine as the control) was added to the dialysis bag (molecular interception of 12,000–14,000; Los Angeles Spectral Medical Industrial Corporation, Los Angeles, CA, USA). Each dialysis bag was suspended in 100 mL of the release medium and slowly stirred in a constant-temperature water bath at 37 ± 1 °C and a rotating speed of 100 r/min. During stirring, 1 mL of the release medium was absorbed at a predetermined time interval and supplemented with 1 mL of the same release medium. The collected samples were centrifuged at 12,000 rpm for 20 min and analyzed using high-performance liquid chromatography (HPLC). Measurements were performed in triplicate.

### 2.6. Efficacy Validation of Zebrafish

#### 2.6.1. Screening of Drug Concentration

The collected embryos (purchased from Feixi, Shanghai, China) were placed in a 24-well plate containing embryonic water and cultured in an artificial climate incubator (RGLC-P160A, Darth Carter, China) at 28 °C. After 24 h post-fertilization (hpf) [26], zebrafish embryos were transferred to embryonic water containing different concentrations of PHL-NCs. The solution was replaced every 12 h, with unscheduled stirring to ensure uniform distribution of drugs. After 72 hpf (48 h exposure) [26], the survival rate and effects of embryos were observed using a stereomicroscope (SRZ-7045DM, COSSim, China) and an inverted fluorescence microscope (Carl Zeiss AG, Oberkochen, Germany).

#### 2.6.2. Transport of NCs in Embryonic Zebrafish In Vivo

The hybrid NCs were used to evaluate NC uptake in zebrafish. Embryos (from 24 hpf to 72 hpf) were exposed separately to PHL-NCs and PHL-NIC-NCs (0.38 mM/L). The embryos treated with embryonic water were set as the blank control group. The embryos were collected at 20 min, 2 h, 6 h, and 12 h, and larvae were collected at 48 h for imaging by an inverted fluorescence microscope (Carl Zeiss AG, Oberkochen, Germany).

#### 2.6.3. Zebrafish Embryo Exposure Experiment

The preliminary experimental operation is shown in Section 2.6.1. After 24 hpf, zebrafish embryos were transferred to embryonic water containing α-arbutin (20 mM/L), PHL-NIC-NCs (0.38 mM/L), and PHL-NCs (0.38 mM/L). Arbutin is commonly used as a common positive control group for melanin inhibition experiments in zebrafish models [20,27,28]. It is a recognized tyrosinase inhibitor with an effective whitening effect [29]. The structure of arbutin is similar to that of tyrosine and competes for tyrosinase and produces competitive inhibition, thus inhibiting tyrosinase activity directly without affecting the mRNA expression of tyrosinase, achieving the effect of inhibiting melanin production [30]. In addition, we found that the optimal concentration of arbutin as a tyrosinase inhibitor was 20 mM/L and the inhibitory effect of α-arbutin was better than that of other arbutin types [19]. Therefore, we used 20 mM/L of α-arbutin as a positive control. After 72 hpf (48 h exposure), the embryos were detached with forceps for observation. The effects of different melanin inhibitors on zebrafish pigmentation were observed by a stereomicroscope (SRZ-7045DM, COSSim, China).

#### 2.6.4. Determination of Tyrosinase Activity in Zebrafish Embryos

The tyrosinase activity of the samples was measured, as described previously with a slight modification [20]. After 48 h exposure, 30 embryos were washed twice with PBS and put into EP tubes containing 300 μL of cold lysate buffer. The tissues were ground and centrifuged at 10,000 r/min for 15 min at 4 °C. Next, 20 μL of the supernatant was added to 96-well plates containing 180 μL of L-DOPA. After incubation at 37 °C in the dark for 30 min, absorbance was measured at 492 nm using a microplate reader (SpectraMax^®^, Molecular Devices, China). The relative tyrosinase activity of the blank control group was recognized as 100%, and the tyrosinase activity of the sample was expressed as the percentage of the blank control group. All the experiments and measurements were performed in triplicate.

#### 2.6.5. Determination of Melanin Content in Zebrafish Embryos

The precipitate obtained by centrifugation in Section 2.6.4 was dissolved in 300 μL of NaOH (1 mol/L), and the EP orifice was sealed with a sealing film. The EP orifice was dried at 95 °C for 30 min, and absorbance was measured at 405 nm. The relative melanin content of the blank control group was considered 100%, and the melanin content of the sample was indicated as the percentage of the blank control group [20].

### 2.7. HPLC Analysis

PHL was detected using an HPLC system (Agilent 1100, Santa Clara, CA, USA) at 280 nm. Separation was performed on an XB-C18 column (Ultimate^®^; 150 × 4.6 mm, 5 μm) with acetonitrile (45:55 *v*/*v*) as the mobile phase, and the flow rate was set as 1 mL/min. In the range of 0.35 to 56 μg/mL, the PHL concentration (C) was linear, with its peak area (A) with a typical calibration curve of C = 0.0155 A–0.1451 and R2 = 0.9998.

### 2.8. Statistical Analysis

All data are presented as the mean ± standard deviation (SD). One-way analysis of variance (ANOVA) using IBM SPSS Statistics 26 (SPSS, Inc., Chicago, IL, USA) was used to compare the survival rate of zebrafish at different concentrations and tyrosinase activity and melanin production after culture with the blank control arbutin, PHL-NCs, and PHL-NIC-NCs. Significance is denoted in the figures as * *p* < 0.05, ** *p* < 0.01, and *** *p* < 0.001.

## 3. Results and Discussion

### 3.1. Characterization of NCs

**Figure 1:**
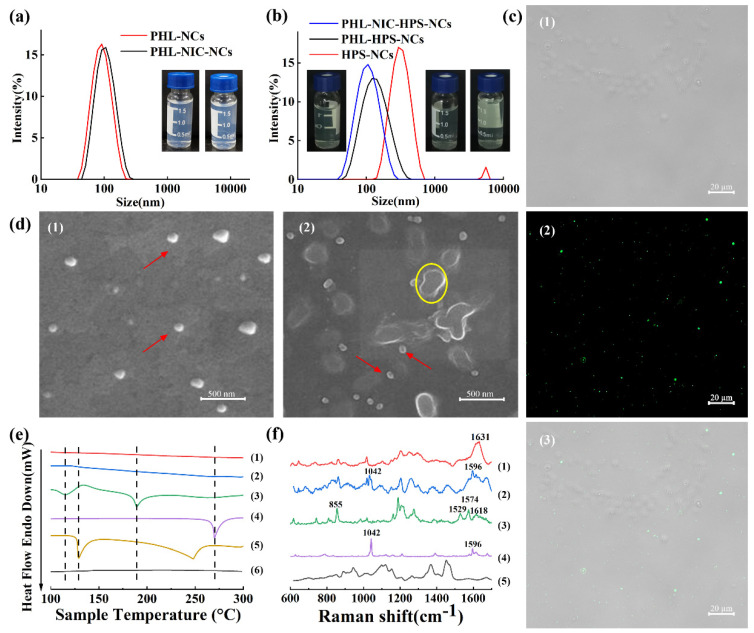
The size distribution and photographs of (**a**) PHL-NCs and PHL-NIC-NCs; (**b**) HPS-NC, PHL-HPS-NC, and PHL-NIC-HPS-NC suspensions; (**c**) (1) brightfield, (2) fluorescence, and (3) merged images of NCs; (**d**) SEM morphologies of (1) PHL-NCs and (2) PHL-NIC-NCs (scale: 500 nm); (**e**) DSC profiles of (1) PHL-NCs, (2) PHL-NIC-NCs, (3) physical mixtures, (4) PHL, (5) NIC, and (6) HPMC; and (**f**) Raman spectra of (1) PHL-NCs, (2) PHL-NIC-NCs, (3) PHL, (4) NIC, and (5) HPMC.

PHL-NCs and PHL-NIC-NCs were successfully prepared by the anti-solvent precipitation method. The particle sizes of PHL-NCs were 91.8 ± 1.0 nm and of PHL-NIC-NCs were 94.6 ± 1.0 nm, while the PDIs were 0.085 ± 0.007 and 0.086 ± 0.003, respectively (Figure 1a). PHL-HPS-NCs, PHL-NIC-HPS-NCs, and HPS-NCs were also prepared by the method described earlier. The particle sizes of PHL-HPS-NCs were 128.1 ± 2.0 nm, of PHL-NIC-HPS-NCs were 95.7 ± 1.0 nm, and of HPS-NCs were 353.3 ± 11.0 nm, while the PDIs were 0.171 ± 0.002, 0.121 ± 0.010, and 0.364 ± 0.046, respectively (Figure 1b). The uncoated, pure NCs of HPS displayed bigger sizes than those displayed by PHL-NCs and PHL-NIC-NCs. The hybrid NCs showed bigger sizes than those showed by the uncoated, pure NCs of PHL and PHL-NIC. This indirectly proves the successful preparation of PHL-HPS-NCs and PHL-NIC-HPS-NCs [31]. The hybrid NCs were detected by an inverted fluorescence microscope (Carl Zeiss AG, Oberkochen, Germany), as shown in Figure 1c. Fluorescence images showed the uniform distribution of hybrid NCs. PHL-NCs displayed a spherical-like morphology (Figure 1d), and the sizes of NCs were comparable to the particle size measurement results. PHL-NCs are indicated by red arrows, and the precipitation after NIC drying is indicated by the yellow circle. 

The DSC profiles of the PHL-NCs, PHL-NIC-NCs, physical mixtures, PHL, NIC, and HPMC are shown in Figure 1e. PHL displayed an endothermal peak at 269.73 °C. NIC showed two endothermal peaks at 129.21 °C and 248.19 °C, while HPMC did not display a typical endothermal peak. The first endothermal peak of the physical mixture was at 115.16 °C, indicating that it relates to metastable eutectic melting, and the second endothermal peak was at 189.17 °C, indicating that this is a characteristic of a mixture capable of cocrystal formation [32]. The endothermal peaks of PHL-NCs and PHL-NIC-NCs disappeared, indicating that the amorphous state was formed. The Raman spectra of the PHL-NCs, PHL-NIC-NCs, PHL, NIC, and HPMC are shown in Figure 1f. PHL [33] was bent as an O–C–C vibration at 855 cm^−1^, a carbon–carbon tensile vibration at 1529 cm^−1^ and 1574 cm^−1^, and a carbonyl C=O stretching vibration absorption peak at 1618 cm^−1^. The NIC characteristic peaks were at 1042 cm^−1^ and 1596 cm^−1^ [34], which are the stretching vibration bands of the -CN amide group and the absorption peak of the deformation vibration of the in-plane ring of the pyridine molecule, respectively. For the PHL-NCs [35], the C=O stretching vibration absorption peak was indicated at 1631 cm^−1^. The characteristic peaks still existed after the preparation of PHL-NIC-NCs. Compared to PHL, the peak of the PHL-NIC-NCs became wider and higher at 1596 cm^−1^ and split into two peaks for NIC at 1042 cm^−1^, changing slightly. This proved that nanocrystals have no effect on the drug structure and do not influence the qualities of PHL and NIC [36]. 

### 3.2. The Stability of NCs

**Figure 2:**
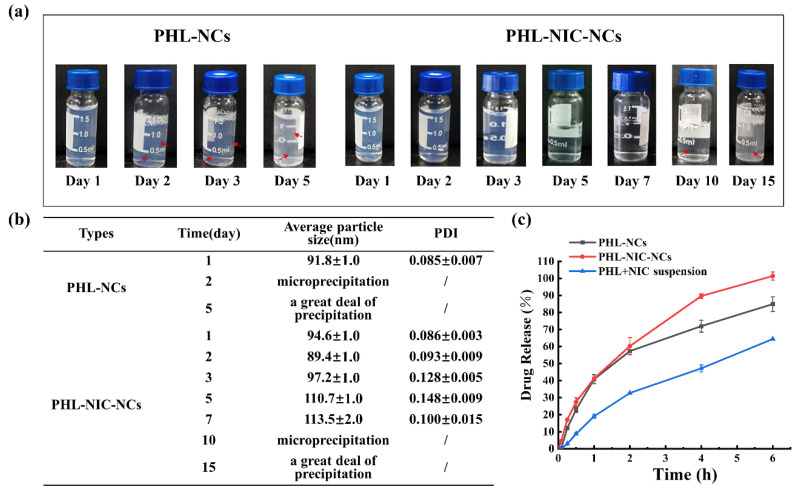
Long-term stability of PHL-NC and PHL-NIC-NC suspensions for (**a**) color changes, (**b**) average particle size and PDI, and (**c**) drug release of PHL and PHL-NIC (*n* = 3).

As shown in Figure 2a, according to the stability test, the PHL-NCs showed a large amount of precipitation on the second day while the PHL-NIC-NCs showed stability without any particle size change for 10 days and large precipitation in 15 days. The suspension color and clarity of the NCs over time are shown in Figure 2b. The PHL-NCs began to appear precipitated (red arrows indicate white precipitated particles) on the second day and gradually changed from a light-blue to a white-turbid suspension as the time increased. The PHL-NIC-NCs did not precipitate until 10 days and changed from a light-blue to a colorless transparent suspension as the time increased and then to a white turbid suspension, which was consistent with the previous particle size and PDI data.

Therefore, we found that PHL-NCs can be stabilized by NIC; the mechanism may be that NIC can interact with PHL through noncovalent interaction and further connect with PHL through hydrogen bonds, while being stabilized by π-π interaction through the phenyl ring between the layers [37].

### 3.3. In Vitro Drug Release Study

The release behaviors of PHL-NC and PHL-NIC-NC suspensions are shown in Figure 2c. It shows that the release of PHL-NCs and PHL-NIC-NCs was faster than that of PHL+NIC powder and the release percentage was only 64.43% ± 0.02% at 6 h, which also indicated that NCs could increase the dissolution rate of insoluble drugs. In addition, the release of PHL-NIC-NCs was faster than that of PHL-NCs, indicating that PHL-NIC-NCs have more advantages in PHL dissolution performance. There may be two possible mechanisms as previously reported, spring and parachute [37]. One is that PHL-NIC-NCs have weaker intermolecular interactions, which facilitates their dissolution [38], and the other is that PHL-NIC-NCs have weaker π-π interaction because the strength of the interaction related to the bond distance between molecules, which can produce higher dissolution rates [39].

### 3.4. Effect of NCs on Melanin Synthesis

#### 3.4.1. Screening of Drug Concentrations

**Figure 3:**
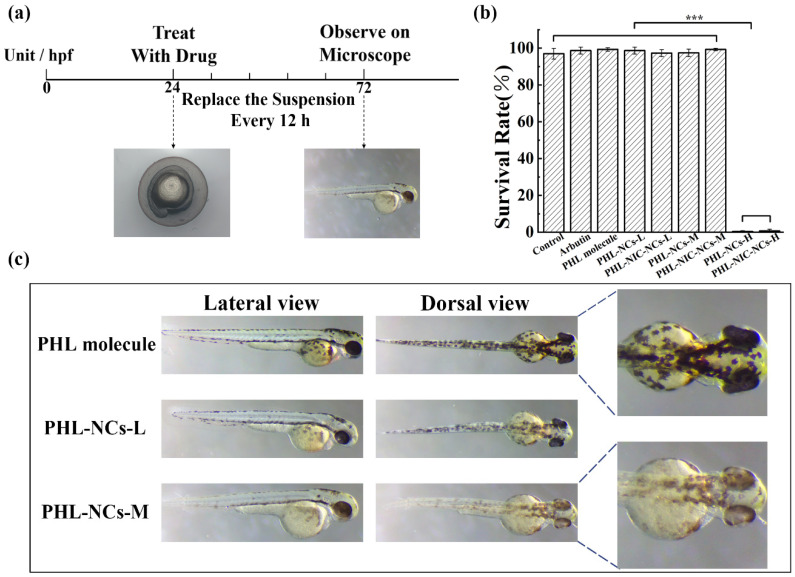
(**a**) Disposition of zebrafish at different stages; (**b**) survival rate of zebrafish cultured with a blank control, arbutin, PHL-NCs, and PHL-NIC-NCs; and (**c**) anti-melanin effect of different concentrations of PHL-NCs on zebrafish. Significance is denoted in the figures as *** *p* < 0.001.

In this section, we screened the concentrations of PHL-NCs and PHL-NIC-NCs in zebrafish. As shown in Figure 3a, 24 hpf embryos were replaced by the suspensions every 12 h until 72 h later to observe zebrafish survival and melanin inhibition. The survival rate experiment of zebrafish included the PHL-NC and PHL-NIC-NC groups, which were set as high (H-0.76 mM/L), medium (M-0.38 mM/L), and low (L-0.19 mM/L)–concentrations, and the PHL molecular group (0.11 mM/L) [35]. The experimental results are shown in Figure 3b. In the high-concentration group (PHL-NCs-H and PHL-NIC-NCs-H), the survival rates of zebrafish were 0.33% ± 0.47% and 0.67% ± 0.94%, respectively, indicating that a large number of deaths occurred. The survival rates of zebrafish in the low-concentration group (PHL-NCs-L and PHL-NIC-NCs-L), the medium-concentration group (PHL-NCs-M and PHL-NIC-NCs-M), and the PHL molecular group were higher than 97%, and there was no significant difference compared to the control group. On this basis, melanin production in zebrafish in the molecular and low- and medium-concentration groups was observed. Figure 3c shows that the medium-concentration group had a better anti-melanogenesis effect than the molecular and low-concentration groups. Therefore, 0.38 mM/L of PHL-NCs and PHL-NIC-NCs was selected as the transport experimental concentration to evaluate the inhibition of melanin production in zebrafish.

#### 3.4.2. Transport of NCs in Embryonic Zebrafish In Vivo

**Figure 4:**
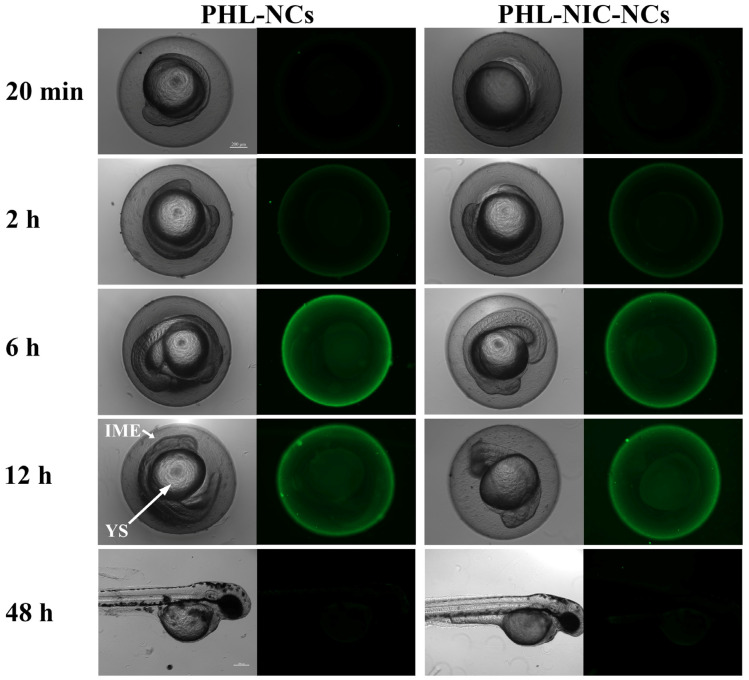
Bright-field and fluorescence images of zebrafish embryos after incubation with 0.38 mM/L of PHL-NCs and PHL-NIC-NCs for 20 min, 2 h, 6 h, and 12 h and zebrafish larvae for 48 h (scale: 200 μm). IME and YS in the figure indicate the inner mass of pancreas and the yolk sac, respectively.

HSP-hybrid PHL-NCs and PHL-NIC-NCs were used to observe the uptake and distribution of intact NCs in zebrafish embryos. As shown in Figure 4, the chorionic surface fluorescence of zebrafish embryos gradually increased as the incubation time increased, indicating the aggregation of intact NCs on the chorion. Fluorescence could also be observed in the inner mass of embryos (IME) and the yolk sac (YS) over time. Interestingly, after 48 h, fluorescence only appeared on the chorion surface and there was no obvious fluorescence in zebrafish larvae, indicating that the NCs entered the chorion in the form of molecules.

#### 3.4.3. Influence of NCs on Tyrosinase Activity and Relative Melanin Content in Zebrafish Embryos

**Figure 5:**
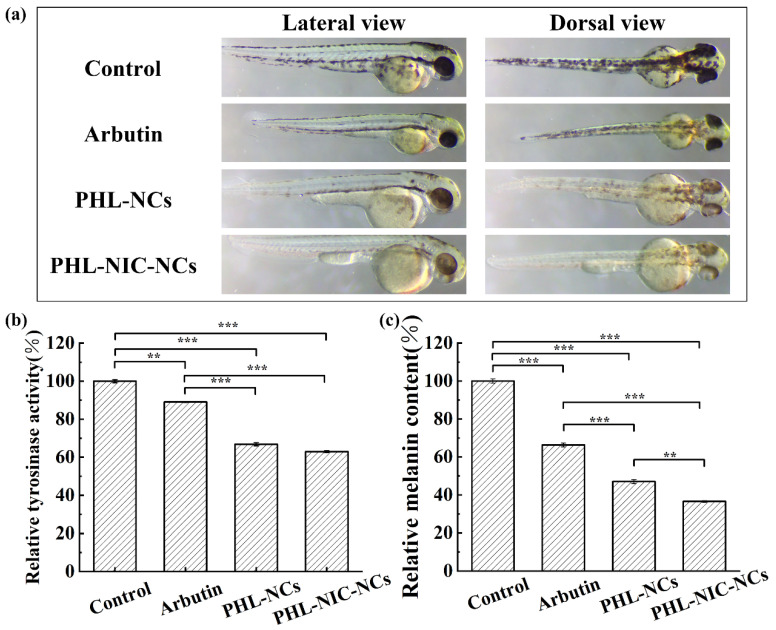
(**a**) Photographs of zebrafish cultured in the blank control group, arbutin (positive control), PHL-NCs, and PHL-NIC-NCs; (**b**) tyrosinase activity; and (**c**) melanin production in zebrafish after culturing with the blank control, arbutin, PHL-NCs, and PHL-NIC-NCs. Significance is denoted in the figures as ** *p* < 0.01, and *** *p* < 0.001.

Embryos at 24 hpf were exposed to various melanogenic inhibitors for 48 h to assess melanogenic inhibitory activity. As shown in Figure 5a, in the blank control group without treatment by inhibitors, a large number of black spots, which were the melanin deposited in the zebrafish embryos, were clearly observed on the lateral and dorsal spine, eyes, and yolk sac of the zebrafish embryos. α-Arbutin was selected as a positive control at 20 mM/L [19]. The results showed that arbutin had little effect on retinal pigment epithelium (RPE) pigmentation but inhibited body pigmentation. Next, we studied the effects of PHL-NCs and PHL-NIC-NCs on zebrafish pigmentation. Two NCs had significant inhibitory effects on body pigmentation in zebrafish, and the inhibitory effect was much better compared to arbutin aqueous solution. This phenomenon may be explained by the fact that NCs increased the diffusion of PHL on the chorion by reducing the particle size of PHL so that the melanin inhibitor could bind more to the enzyme site [23], thereby improving bioavailability. The inhibitory effect of PHL-NIC-NCs on zebrafish melanin was better than that of PHL-NCs. For PHL-NCs, a small amount of lighter melanin was found in the yolk sac and on the top of the spine. For PHL-NIC-NCs, only a small amount of lighter melanin was deposited on the top of the spine and melanin inhibition in other parts was effective. In addition, we found that two NCs had an inhibitory effect on RPE pigmentation in zebrafish. The process of zebrafish pigmentation is different from that of RPE pigmentation, and its formation may be regulated by Tyr, Tyrp1, and Dct/Trp2 genes. The affinity of drugs or preparations to RPE or RPE-associated melanosomes may also impact their effects, but the effects of PHL-NCs and PHL-NIC-NCs on RPE pigmentation need further studies to prove.

To further evaluate the anti-melanogenic activity of PHL-NCs and PHL-NIC-NCs, their effects on the reduction in tyrosinase activity (Figure 5b) and melanin content (Figure 5c) in zebrafish embryos were investigated. The tyrosinase activity and melanin content decreased to 66.85% ± 0.87% and 47.04% ± 1.02%, respectively, after treatment with 0.38 mM/L of PHL-NCs. After treatment with 0.38 mM/L of PHL-NIC-NCs, the tyrosinase activity and melanin content decreased to 62.97% ± 0.52% and 36.57% ± 0.44%, respectively. It can be highlighted that PHL-NCs and PHL-NIC-NCs showed more significant inhibition of tyrosine activity (66.85% ± 0.87% and 62.97% ± 0.52%) than the positive control arbutin (89.04% ± 0.26%) and a more obvious anti-melanogenesis effect (47.04% ± 1.02% and 36.57% ± 0.44%) than arbutin (66.33% ± 1.03%). According to the experimental results of the tyrosinase activity and melanin content in zebrafish, there was no significant difference in the inhibitory effect of tyrosinase activity between PHL-NCs and PHL-NIC-NCs, indicating that NIC does not increase the inhibitory effect of PHL. However, for the inhibition of melanin content formation, the PHL-NIC-NC group showed significant improvement in anti-melanin performance, indicating that NIC reduces melanin content through synergy. 

**Figure 6:**
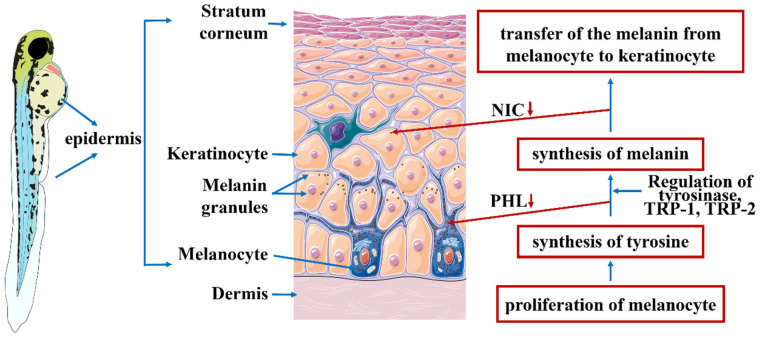
The mechanism of PHL and NIC inhibiting melanin production in zebrafish.

As shown in Figure 6, the melanin generation process is divided into three stages: proliferation of melanin, synthesis of tyrosine and melanin, and transfer of melanin from melanocytes to keratinocytes [40]. Tyrosinase activation is required when tyrosine is converted into melanin [41]. PHL and NIC act on stage 3 and stage 4, respectively. The mechanism of PHL is similar to that of arbutin, but PHL-NCs suppress the activity of tyrosinase in the first step of the rate-limiting enzyme in melanin synthesis, which causes conformational changes to the enzyme after binding to tyrosinase, thus reducing melanin production [10]. The mechanism of NIC is completely different with three main mechanisms [6,42,43]: interfering with keratinocyte and melanocyte interactions, inhibiting the transfer of the generated melanin, and promoting melanin transfer to the cuticle and promoting cuticle shedding.

## 4. Conclusions

The formation of melanin involves mainly two pathways. We combined the inhibition of tyrosinase activity with the inhibition of melanin transfer by fabricated PHL-NIC-NCs and evaluated the anti-hyperpigmentation effect in a larval zebrafish model. This study systematically investigated the preparation and characterization of PHL-NCs and PHL-NIC-NCs and administered them to zebrafish to evaluate the tyrosinase inhibitory and anti-melanogenesis abilities of NCs. In addition, we studied PHL-NIC-NCs using the HPS fluorescence marker to assess the uptake and distribution of NCs in zebrafish. The results indicated that PHL-NIC-NCs increase the inhibition effect of tyrosinase activity and melanogenesis. Furthermore, anti-melanin efficacy can be enhanced by a combination of PHL-NCs and NIC.

NC solve many problems of existing preparations (including nanoemulsions, lipid NPs, chitosan NPs, and polymer NPs), such as low drug loading, high production costs and safety, and improved absorption efficiency and bioavailability. In addition, we found that the combined use of two anti-melanin drugs, PHL and NIC, not only improves stability and solubility but also improves bioavailability and absorption efficiency so as to have more anti-melanin efficacy and produce higher value. This research will provide a reference for the future development of anti-melanin efficacy and the combined use of drug formulations.

However, this direction still needs further analysis, such as how to understand whether NIC increases the stability of PHL-NCs by increasing surface charge through zeta potential analysis so as to explore more mechanisms of NIC-stabilizing PHL-NCs. In addition, for this kind of preparation, we only hope that the drug can stay in the skin’s basal layer and stay in the blood as little as possible. However, more animal models are still needed to verify this aspect, which will be the direction of future research.

## Data Availability

Not applicable.

## Abbreviations

HPMC	Hydroxypropyl methylcellulose
NCs	Nanocrystals
PHL	Phloretin
NIC	Nicotinamide
HPS	1,1,2,3,4,5-Hexaphenylsilole
PHL-NCs	Phloretin-nanocrystals
PHL-NIC-NCs	Phloretin-nicotinamide-nanocrystals
PHL-HPS-NCs	Phloretin-1,1,2,3,4,5-hexaphenylsilole-nanocrystals
PHL-NIC-HPS-NCs	Phloretin-nicotinamide-1,1,2,3,4,5-hexaphenylsilole- nanocrystals
PDI	Polydispersity index
SC	Stratum corneum
NPs	Nanoparticles
DDSs	Drug delivery systems
dpf	Days post-fertilization
hpf	Hours post-fertilization
AIE	Aggregation-induced emission
HPLC	High-performance liquid chromatography
SD	Standard deviation
ANOVA	Analysis of variance
PHL-NCs-L	Phloretin-nanocrystals-low
PHL-NCs-M	Phloretin-nanocrystals-medium
PHL-NCs-H	Phloretin-nanocrystals-high
IME	Inner mass of embryos
YS	Yolk sac
RPE	Retinal pigment epithelium
